# Biomechanical Comparisons of Two Types of Tuina in Treating Lumbar Disc Herniation: A Finite Element Analysis

**DOI:** 10.1155/prm/6643204

**Published:** 2026-02-27

**Authors:** Frank Fan Huang, Ming-wang Qiu, Wan-ming Wu, Jia-jun Liu, Qingkai Zhao, Si-yi Zhao, Man-qi Lu, Zhao-xian Yan, Jia-qi Li, Xian Liu, Yi-kai Li, Arnold Yu Lok Wong, Zhi-yong Fan

**Affiliations:** ^1^ Department of Rehabilitation Sciences, The Hong Kong Polytechnic University, Hung Hom, Kowloon, Hong Kong Special Administrative Region, China, polyu.edu.hk; ^2^ The Second Affiliated Hospital of Guangzhou University of Chinese Medicine, Yuexiu District, Guangzhou, China, gzucm.edu.cn; ^3^ The First Clinical Medical College of Guangzhou University of Chinese Medicine, Baiyun District, Guangzhou, China, gzucm.edu.cn; ^4^ Tianjin Hospital, Tianjin University, Heping District, Tianjin, China, tjorthop.org; ^5^ Department of Mechanical Engineering, The Hong Kong Polytechnic University, Hung Hom, Kowloon, Hong Kong Special Administrative Region, China, polyu.edu.hk; ^6^ The Fifth People’s Hospital of Longgang District, Longgang District, Shenzhen, China; ^7^ Department of Rehabilitation Medicine, Shenzhen Hospital, Southern Medical University, Baoan District, Shenzhen, China, fimmu.com; ^8^ The Third Affiliated Hospital of Southern Medical University, Tianhe District, Guangzhou, China, fimmu.com

**Keywords:** finite element analysis, high-speed oblique Tuina, low-speed oblique Tuina, lumbar disc herniation

## Abstract

**Background:**

Prior research has demonstrated the clinical efficacy of two specialized Tuina techniques, namely, high‐speed oblique Tuina (HSOT) and low‐speed oblique Tuina (LSOT), in the treatment of lumbar disc herniation (LDH). These techniques integrate principles of traditional Chinese medicine with modern manual therapy. Despite their proven therapeutic benefits, the biomechanical effects of HSOT and LSOT on spinal structures remain poorly understood.

**Aim:**

Finite element analysis (FEA) was utilized to investigate the biomechanical effects of HSOT and LSOT on a patient with LDH, aiming to elucidate their underlying treatment mechanisms.

**Methods:**

The mechanical parameters of HSOT and LSOT applied to a participant with left L4/5 LDH were meticulously measured using a state‐of‐the‐art Multipoint Thin Film Pressure Testing System. These comprehensive data, along with the corresponding high‐resolution computerized tomography (CT) scan data, were subsequently input into four advanced finite element analysis (FEA) software programs. These programs were employed to conduct a detailed analysis of the stress–strain characteristics of both HSOT and LSOT on the spinal structures, enabling a thorough comparison of their biomechanical effects and potential therapeutic outcomes.

**Results:**

The maximum stress and average displacement of vertebral and ligamentous structures during HSOT in the lumbar vertebrae‐pelvis model doubled those of LSOT. HSOT caused the maximum average displacement of the L4/5 right intertransverse ligament, whereas LSOT induced the maximal mean displacement of the L4/5 left intertransverse ligament. Additionally, HSOT caused the maximum average displacement of the right iliolumbar ligament, but the maximum mean displacement was observed in the left iliolumbar ligament during LSOT.

**Conclusion:**

Both HSOT and LSOT produced small displacements of the lumbar and pelvic structures, but HSOT elicited significantly greater displacement and stress on various spinal tissues than LSOT. These mechanical responses may be the biomechanical effects of HSOT and LSOT in people with LDH.

**Trial Registration:** ClinicalTrials.gov identifier: ChiCTR2200065450

## 1. Introduction

Lumbar disc herniation (LDH) is one of the spinal diseases, which is manifested as a proportion of the disc protruding into the surrounding space [1]. Approximately 95% of LDH cases occur at the L4‐5 and L5‐S1 levels [2], which may be a potential etiology of low back pain (LBP) [3]. LDH‐related LBP has imposed tremendous economic and medical burdens on individuals and societies alike [4]. Conservative treatments are commonly used to reduce pain and improve physical function in patients with LDH [5–7]. However, nonsteroidal anti‐inflammatory drugs (NSAIDs), the first‐line analgesics for LBP and sciatica, can cause adverse effects [8], such as upper gastroenteritis [9]. Lately, traditional Chinese medicine, as a part of conservative treatments, has been gaining its popularity for treating patients with LDH [10], among which Tuina has demonstrated good effectiveness in treating LDH [11]. In southern China, clinicians and physicians usually use high‐speed oblique Tuina (HSOT) and low‐speed oblique Tuina (LSOT) to treat patients with LDH, and the usual clinical practice involves one HSOT and three LSOT [12]. The clinical efficacy of HSOT and LSOT has been demonstrated in prior research [13]. These techniques are similar to combining mobilization and manipulation used by physiotherapists. Scholars examined how different manual therapy techniques affect spinal loads by analyzing spinal tissue load and surface pressure curves [14], and cadaver experiments fail to capture internal stress changes inside human structures.

With the advancement of computer technology, the finite element analysis (FEA) of spine biomechanics has become a useful research tool to estimate spine biomechanics [15]. The lumbar finite element model (FEM) derives its data directly from medical imaging, such as computerized tomography (CT). Different motion states of the spine can be simulated by changing the boundary and load conditions, like changing the material properties of the model to simulate the pathological changes of the spine, enabling medical personnel to better understand the biomechanical pathogenesis of LDH [16–18]. Further, it can simulate and estimate the biomechanical characteristics of the lumbar intervertebral disc under various conditions, so it has been widely used in the LDH‐related biomechanical research [19].

This approach aims to estimate the biomechanical mechanisms underlying HSOT and LSOT by modeling between the therapeutic modalities and the affected spinal structures. Given the above, the current research used an FEA to investigate the biomechanical effects of HSOT and LSOT on an individual with LDH in order to estimate the biomechanical mechanisms of these treatments.

## 2. Materials and Methods

### 2.1. CT and Magnetic Resonance Imaging (MRI) Scan Data Acquisition and the Development of an FEM

The current study was approved by the Institutional Ethics Committee of The Second Affiliated Hospital of Guangzhou University of Chinese Medicine (No. YF 2022‐115‐01) and was registered in the Chinese Clinical Trial Registry (ChiCTR2200065450). The data were obtained from a 27‐year‐old male volunteer (170 cm in height, and 60 kg in weight) with unilateral LDH on the left side. The participant had no history of spine surgery, osteoporosis, or spinal fractures. He underwent lumbopelvic CT scans in a 128‐slice CT scanner (Siemens, Munich, Germany). The transverse scanning was performed on the whole lumbar spine and the pelvis with 0.5 mm slices. He also underwent lumbopelvic magnetic resonance imaging (MRI) scan in a 3.0 Tesla (T) MRI scanner (Philips, Amsterdam, the Netherlands). His CT and MRI scan showed a left posterolateral intervertebral disc protrusion at the L4/5 level. According to the guidance of the Chinese Pain Expert Consensus on Diagnosis and Treatment of Lumbar Disc Herniation issued by the Chinese Medical Association [20] and Pfirrmann systems [21], he was diagnosed with an LDH at Pfirrmann grade III level.

To construct a three‐dimensional (3D) FEM of the lumbar‐pelvis region, the participant’s CT data in the DICOM format were imported to Mimics 21.0 (Materialise Company, Belgium). The three‐dimensional (3D) data in Mimics 21.0 were presented in *XYZ* axes, where the *x*‐axis is the coronal axis, the *y*‐axis is the sagittal axis, and the *z*‐axis is the vertical axis. The software program extracted and transformed the data into an original 3D model. All surface models were meshed using Geomagic Warp 2017 (Raindrop Company, USA). Then, we imported the optimized and retouched surface solids in STP format into Solidworks 2017 software (Dassault Systemes, France) for assembly. Finally, the material properties, boundary conditions, coordinates, and loading settings for the model were added, and the FEA was performed using Ansys Workbench 17.0 (Ansys Company, USA). The modeling approach in this study adopted the tetrahedral meshing methodology developed by Zhang et al. [22], where CT and MRI data from LDH patients were utilized. Specifically, CT data were employed for bone modeling, while MRI data were used for diagnosing. Based on the measurement methods described in the literature, the participant’s thickness of endplate was set as 0.1 mm and his cortical bone was set as 0.4 m [23]. His nucleus pulposus accounts for about 41% of the total volume of the intervertebral disc [24, 25]. The model was meshed in ANSYS using 0.5 mm surface elements for cancellous bone, cortical bone, and ligaments, while shared nodal surfaces at the nucleus periphery generated 0.25 mm meshes for both the annulus fibrosus and nucleus pulposus. For boundary conditions and material assumptions, the nucleus pulposus, the annulus fibrosus, and all bony tissues were considered isotropic, homogeneous, elastic materials, and ligaments were simulated using 1D nonlinear spring elements to ensure computational accuracy. The lumbar ligaments included the articular capsule (AC), anterior longitudinal ligament (ALL), posterior longitudinal ligament (PLL), ligamentum flavum (LF), intertransverse ligament (ITL), interspinal ligament (IL), and supraspinous ligament (SL). Ligaments of the pelvis consist of the pubic ligament (PL), anterior sacroiliac ligament (ASL), posterior sacroiliac ligament (PSL), iliolumbar ligament (IL), sacrospinous ligament (SS), and sacrotuberous ligament (ST) [26]. Each of these structures was modeled as a 3D tension‐only truss component. The relevant ligament attachment sites and the corresponding cross‐sectional area were added to the model as suggested in the literature [26]. The facet joints were set as a nonlinear 3D contact, with the surface‐to‐surface contact elements. The posterior structures and articular cartilage were defined as a frictional contact with a friction coefficient of 0.1 [27]. The 3D lumbar‐pelvic FEM consists of a total of 344,521 elements and 550,539 nodes. Its reliability was validated using the previous method [28, 29]. The material properties of each structural organization are summarized in Table 1 [30, 31].

**TABLE 1:** Material properties of the finite element model.

Number	Materials	Elastic modulus (MPa)	Poisson ratio	Cross‐sectional area (mm^2^)
1	Cortical bone	11520	0.2	—
2	Cancellous bone	126	0.3	—
3	Endplate	32	0.25	—
4	Anulus fibrosus	92	0.45	—
5	Nucleus pulposus	1	0.49	—
6	Posterior elements	3500	0.3	—
7	Articular cartilage	35	0.4	—
8	Anterior longitudinal ligament	20	0.3	63.7
9	Posterior longitudinal ligament	70	0.3	314
10	Ligamentum flavum	50	0.3	314
11	Interspinal ligament	28	0.3	30
12	Supraspinous ligament	28	0.3	314
13	Intertransverse ligament	58.7	0.3	3.6
14	Pubic ligament	10	0.3	30
15	Posterior sacroiliac ligament	13.3	0.2	100
16	Anterior sacroiliac ligament	20.8	0.2	314
17	Iliolumbar ligament	30	0.3	314
18	Sacrotuberous ligament	50	0.3	50
19	Sacrospinous ligament	12.5	0.3	30

### 2.2. Mechanical Force Measurement

**FIGURE 1:**
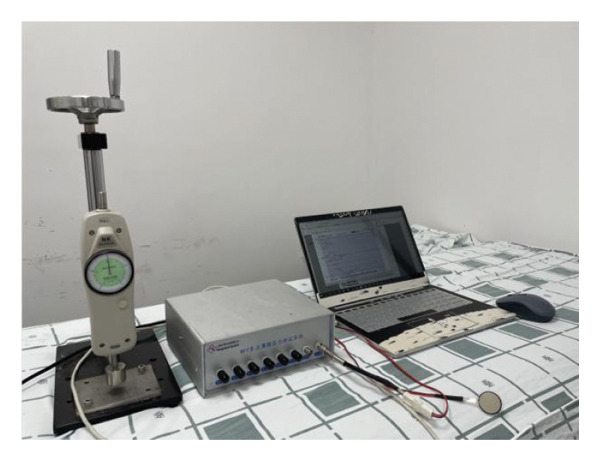
MFF multipoint thin‐film pressure‐testing system.

To measure the pressure applied to the spine through the therapist’s hand during HSOT and LSOT, a Multipoint Thin Film Pressure Testing System (Yicheng company, Shanghai, China) with one type 401 pressure film sensor (thickness 0.2 mm, length 67 mm, width 33 mm, sensing area diameter 25.4 mm) was used [32]. The amplification of the multipoint membrane pressure test system was set at a value of 7. The output voltage was maintained at 3–4 V, and the range of sensitivity was set at 0.1 N. Then, we used a calibration station, which was collocated with the Multipoint Thin Film Pressure Testing System, to calculate the fitting function *y* = *kx* + *b* (where *y* is force and *x* is voltage). We first put the one type 401 pressure film sensor under the Multipoint Thin Film Pressure Testing System and then applied a sequence of force increments using an adjustable pressure iron cylinder that was precisely set at intervals of 10, spanning the range from 0 to 50, denoted as “*y*”. Therefore, we obtained the relevant voltage values, denoted as “*x*”. After the calibration, the calculated *k* value was 11.861 and the *b* value was 0.4868. These two values were then entered into the acquisition software, and the data acquisition frequency was set at 50 Hz. The values measured by the test system were the actual pressure values, and the stress range was between 100 N and 800 N. We collected the average impact force, impact time, and return wave during the entire period of HSOT and LSOT, and they were used in the subsequent FEA. The setup of the Multipoint Thin Film Pressure Testing System is shown in Figure 1.

### 2.3. Manipulation of HSOT and LSOT

**FIGURE 2:**
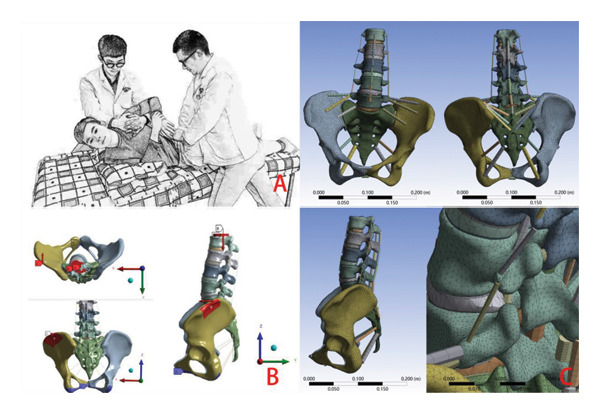
Images of the delivery of the high‐speed oblique Tuina, and the finite element analog simulation images. The red dot represents the location of the force application, while the red arrow indicates the direction of the applied force.

A manual therapist with more than 10 years of experience delivered the treatment. The participant initially lay on his right side with a pillow placed under his right shoulder to elevate the upper body. His right upper hand was used to pull the left wrist, bringing both arms close to the chest. Meanwhile, his legs were straightened and manually separated by 30° by the therapist to prerotate the L4‐5 level at the hypothetical shear center [33]. After the body was placed in this position, an assistant stabilized the participant’s shoulders so that the line between the two shoulders was perpendicular to the bed surface. The therapist then positioned his palms on the participant’s left iliac wing, placing a pressure film sensor between his hand and the participant’s skin. HSOT: Gradually, the therapist applied downward pressure to push the participant’s abdomen toward the bed surface, allowing the participant’s trunk to passively rotate to its end range based on the therapist’s palpation of the end feel. Subsequently, the therapist delivered a single high‐speed, low‐amplitude downward thrust to the joint, slightly exceeding the physiological range of trunk rotation. LSOT: Following this, the therapist then delivered the LSOT by pressing the palms on the participant’s iliac wing as before. Notably, the therapist pushed the iliac wing slowly and slightly until he felt the resistance of the soft tissue around the joint and felt the end range, and subsequently, a low‐speed, low‐amplitude thrust was applied to the joint, inducing a deliberate augmentation of joint rotation beyond its physiological range [12] break period was observed between delivering HSOT and LSOT. The same procedure for both HSOT and LSOT was performed by the therapist three times, with a 30‐min break between each session. To ensure greater accuracy of the mechanical values, the resulting impact force, impact time, and return wave during HSOT and LSOT were averaged (Figure 2A). The purpose of our separate measurement of three HSOT operations and three LOST operations is to measure the average mechanical parameters of the two manipulation operations, providing parameters for the finite element research in the following text.

### 2.4. Estimating Spinal Mechanical Responses During HSOT and LSOT Using FEMs

The distinct mechanical responses of the lumbar spine during HSOT and LSOT were evaluated by an FEA that followed the biomechanics analysis suggested by Bi et al. using the mechanical test results collected from the pressure testing system [34]. Specifically, to simulate the two treatment conditions, the lower surface of the ischial bone was fixed. An axial rotational moment of 15 N·m was applied to the L1 upper endplate to simulate the preparatory posture preceding oblique manipulation. Additionally, a compressive load of 150 N was applied to simulate tension within muscles, ligaments, and other related structures. Then, we applied the pressure acquired from the pressure testing system to the left lateral area of the left iliac wing spinous process and precisely targeted it in a direct anterior direction to generate a counterclockwise rotation force (the anterior direction is the participant’s sagittal axis, the *x*‐axis is the coronal axis, the *y*‐axis is the sagittal axis, and the *z*‐axis is the vertical axis) (Figure 2B). The loads of HSOT and LSOT were entered into the respective models according to the mechanical data measured by the pressure testing system. According to our clinical practice one HSOT and three LSOT [12], the average impact force, impact time, and return wave trough time of the average three tests of HSOT and LSOT was entered into the FEM to explicitly match the usual clinical practice. The contact area of both HSOT and LSOT was 10 cm^2^.

## 3. Results

### 3.1. Collection of Mechanical Parameters

#### 3.1.1. The Time–Stress Curves

**FIGURE 3:**
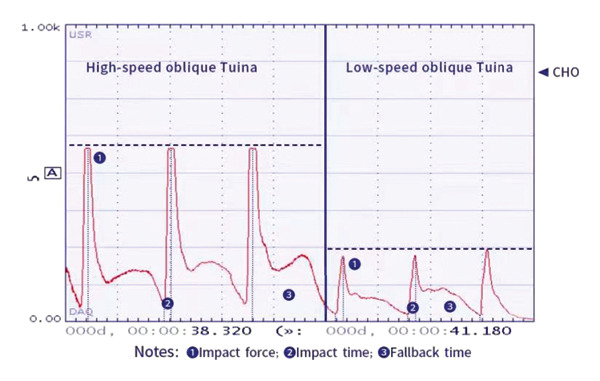
The time–stress curves of high‐speed oblique Tuina and low‐speed oblique Tuina.

The impact force (N), impact time (s), and fallback time (s) of HSOT and LSOT were used to plot the time–stress curves (Figure 3).

#### 3.1.2. The Mechanical Parameters of HSOT and LSOT

The average impact force of HSOT was 580 N, and the mean impact time was 0.15 s, while the return wave trough time was 1.5 s. Conversely, the average impact force of LSOT was 220 N, the impact time was 0.3 s, and the return wave trough time was 1.2 s. The mechanical parameters of HSOT and LSOT are illustrated in Table 2.

**TABLE 2:** Mechanical parameters of high‐speed oblique Tuina (HSOT) and low‐speed oblique Tuina (LSOT).

Operation	Average impact force (N)	Impact time (s)	Return wave trough time (s)
HSOT 1	595.8	0.14	1.5
HSOT 2	575.9	0.15	1.6
HSOT 3	568.3	0.16	1.4
Average value of HSOT	580	0.15	1.5
LSOT 1	226.1	0.32	1.2
LSOT 2	219.3	0.26	1.1
LSOT 3	214.6	0.28	1.3
Average value of LSOT	220	0.30	1.2

### 3.2. FEM Control Equations and Numerical Simulation Methodology

Given the complex biomechanical interactions involved in manipulation techniques, this study employs a finite element model based on Hooke’s law to accurately characterize the mechanical evolution of the system [35]. Specifically, the generalized Hooke’s law is applied to account for three‐dimensional stress and strain states, enabling precise simulation of the model’s elastic modulus behavior.

### 3.3. Validation of the FEM

#### 3.3.1. Grid Independence Verification

In order to verify the effectiveness of the mesh division method used in this study, and to obtain the best balance between computational accuracy and computational resources, the basic finite element model was tested for mesh convergence, and eight mesh schemes were generated according to the element size of the model, which were automatically divided into 5.0, 4.5, 4.0, 3.5, 3.0, 2.5, and 2 mm, respectively, for irrelevance verification. The total number of grids corresponding to different schemes is shown in Table 3.

**TABLE 3:** Different grid division schemes.

Grid independence verification	Node	Grid	Overall stress results (MPa)
Automatic system division	130938	66791	26.256
Mesh size 5.0 mm	154792	81435	22.188
Mesh size 4.5 mm	178406	94423	24.264
Mesh size 4.0 mm	211100	112545	31.34
Mesh size 3.5 mm	260313	140099	34.12
Mesh size 3.0 mm	332153	180262	28.603
Mesh size 2.5 mm	450986	247121	27.024
Mesh size 2.0 mm	675987	375880	27.534

When the pubic symphysis was constrained, a rotational moment (15 N ∗ m) and a vertical downward force (150 N) were applied to the upper surface of the L1 vertebral body for testing. The maximum von Mises stress of different tissues in the finite element model was calculated and compared. The meshing was considered convergent when the difference between the predictions of the two meshing schemes compared was less than 5%. The stress results are shown in Table 3.

When the element size is automatic, the stress result is 26.256 MPa, and when the element size is 2.5 mm, the stress result is 27.024 MPa. The calculated difference between the two is 2.84%, which meets the requirements of mesh convergence test. Therefore, a 2.5‐mm element size was used in the simulation mesh scheme of the FE model carried out in this study, considering the computational cost comprehensively.

#### 3.3.2. Validation of the Simulation Method

The loading boundary conditions applied to the current lumbar pelvic model were akin to those utilized in previous studies [28, 29]. Specifically, the lumbar range of motion of our FEM (i.e., flexion, extension, lateral flexion, and axial rotation) was aligned closely with that observed in prior research [28, 29]. The successful development of the lumbar pelvis FEM in this study highlights its potential for future modeling and analysis purposes. The validation results of our FEM are illustrated in Table 4.

**TABLE 4:** Validation of finite element model.

Study name	Forward bending range of motion	Backward extension range of motion	Left and right lateral bending range of motion	Axial rotation activity range
Wen et al. [28]	7.84°		7.25°	3.88°
Song et al. [29]	10.56°		11°	4.75°
Renner et al. [36]	12.13°		9.82°	4.46°
This study	7.53°	4.63°	9.8445°	5.1921°

### 3.4. Stress and Displacement Results of the Overall Model Using Spinal Mechanical Responses During HSOT and LSOT

The highest estimated stress in the lumbar spine‐pelvic model was located at the median sacral crest, with a stress of 58.68 MPa and an average overall displacement of 0.9621 mm. Further, the average displacement of the overall model on the *X*, *Y*, and *Z* axes was 0.0779 mm, −0.8400 mm, and 0.0366 mm, respectively, where the negative sign represents the opposite direction. During LSOT, the lumbar‐pelvis model exhibited a maximum stress of 32.2840 MPa, which was located at the median sacral crest. The average overall displacement was 0.3327 mm, while the average deformations of the entire lumbar region along the *X*, *Y*, and *Z* axes were 0.0423 mm, 0.1529 mm, and −0.7090 mm, respectively (with the negative sign indicating the opposite direction). The comparison between the maximum stress and average displacement of the overall model during HSOT and LSOT is shown in Figure 4.

FIGURE 4Comparisons between the maximum stress and average displacement of the finite element models using high‐speed oblique Tuina (HSOT) and low‐speed oblique Tuina (LSOT). Notes: (A) shows the maximum stress of the finite element model during HSOT; (B) shows the average displacement of the finite element model during HSOT; (C) shows the maximum stress of the finite element model during LSOT; (D) shows the average displacement of the finite element model during LSOT.

**Figure:**
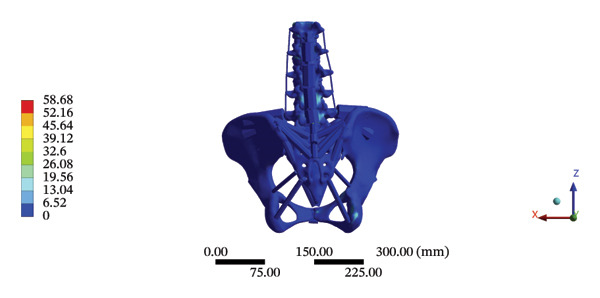
(A)

**Figure:**
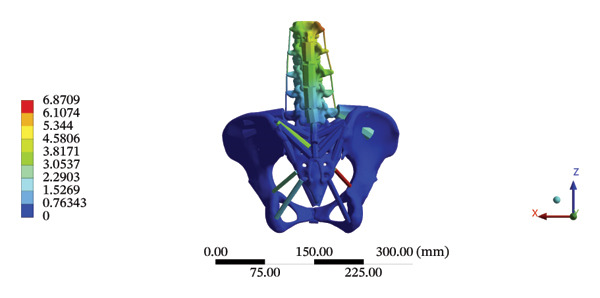
(B)

**Figure:**
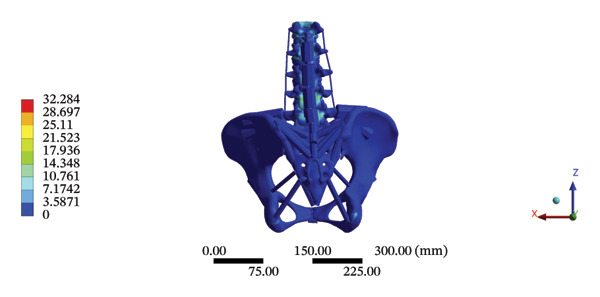
(C)

**Figure:**
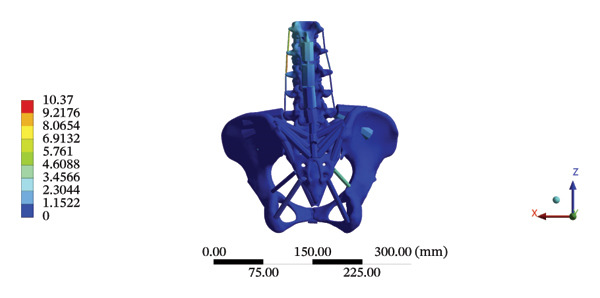
(D)

### 3.5. Bone Structure Models

#### 3.5.1. Lumbar Biomechanics

Although the loading forces were applied to the right iliac wing, the force indirectly induced lumbar spine movement. Our model showed that the maximum stress was found at the right L4/5 articular cartilage (11.1090 MPa) during HSOT, while the maximum average displacement was found at the right L1/2 articular cartilage (4.4531 mm). The stress and average displacement of other lumbar segments are shown in Supporting Table S1. Likewise, the maximum stress was located at the left L1/2 articular cartilage (11.0820 MPa) during LSOT, whereas the maximum average displacement of the left L4/5 articular cartilage was 2.5362 mm. The stress and average displacement changes of other lumbar segments are shown in Supporting Table S2. The graphical comparisons of the maximum stress and average displacement of the lumbar spinal structure during HSOT and LSOT are shown in Figures 5 and 6.

FIGURE 5Comparison between the maximum stress and average displacement of the cortical and cancellous bone using high‐speed oblique Tuina (HSOT) and low‐speed oblique Tuina (LSOT). (A) Maximum stress of the cortical bone during HSOT; (B) average displacement of the cortical bone during HSOT; (C) maximum stress of the cancellous bone during HSOT; (D) average displacement of the cancellous bone during HSOT; (E) maximum stress of the cortical bone during LSOT; (F) average displacement of the cortical bone during LSOT; (G) maximum stress of the cancellous bone during LSOT; and (H) average displacement of the cancellous bone during LSOT.

**Figure:**
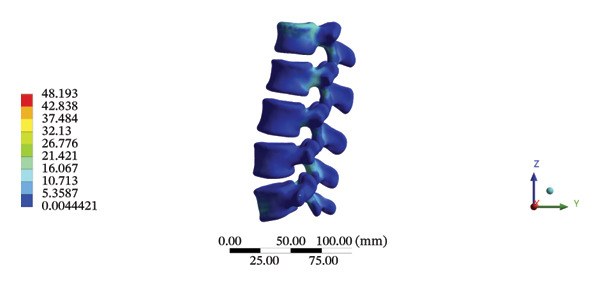
(A)

**Figure:**
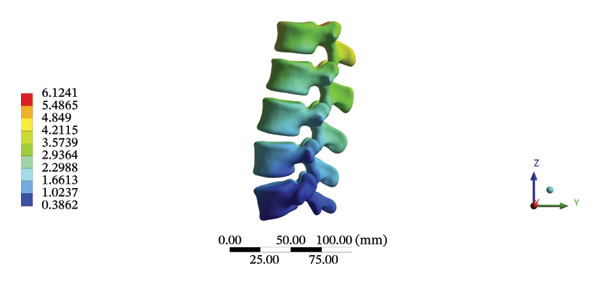
(B)

**Figure:**
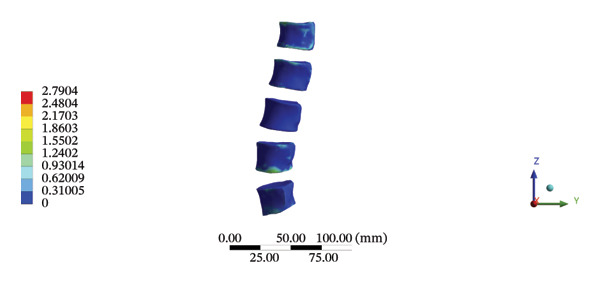
(C)

**Figure:**
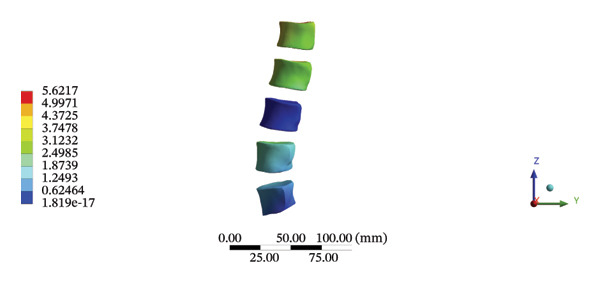
(D)

**Figure:**
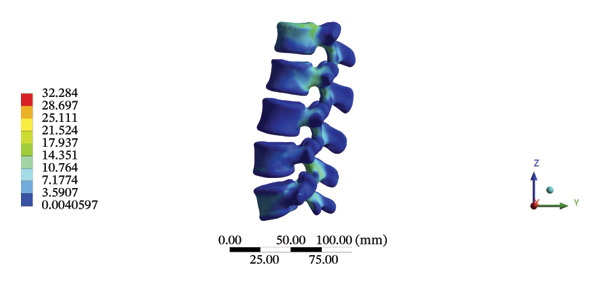
(E)

**Figure:**
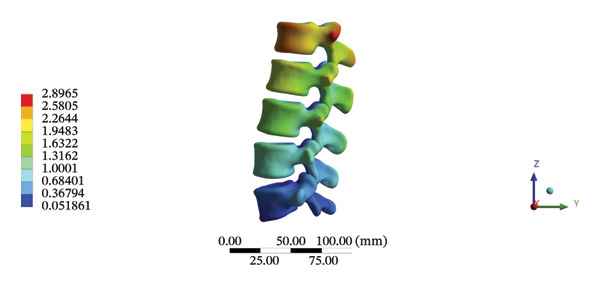
(F)

**Figure:**
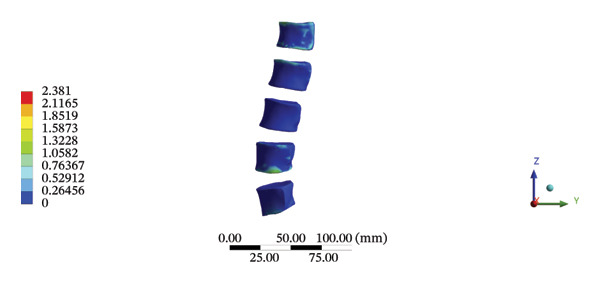
(G)

**Figure:**
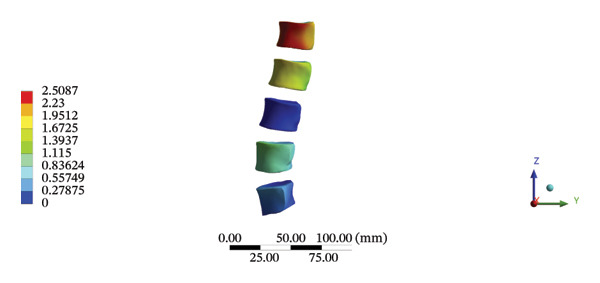
(H)

FIGURE 6Comparison between the maximum stress and average displacement of the articular cartilage and endplate using high‐speed oblique Tuina (HSOT) and low‐speed oblique Tuina (LSOT). Notes: (A) shows maximum stress of the articular cartilage during HSOT; (B) shows average displacement of the articular cartilage during HSOT; (C) shows maximum stress of the endplate during HSOT; (D) shows average displacement of the endplate during HSOT; (E) shows maximum stress of the articular cartilage during LSOT; (F) shows average displacement of the articular cartilage during LSOT; (G) shows maximum stress of the endplate during LSOT; (H) shows average displacement of the endplate during LSOT.

**Figure:**
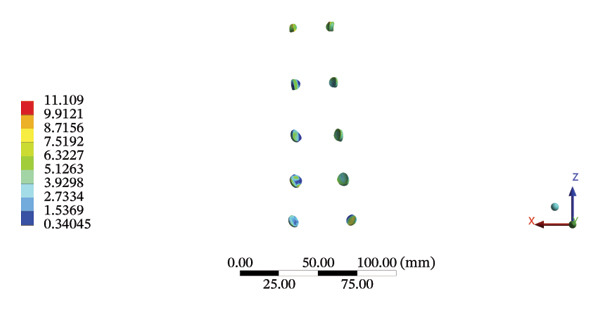
(A)

**Figure:**
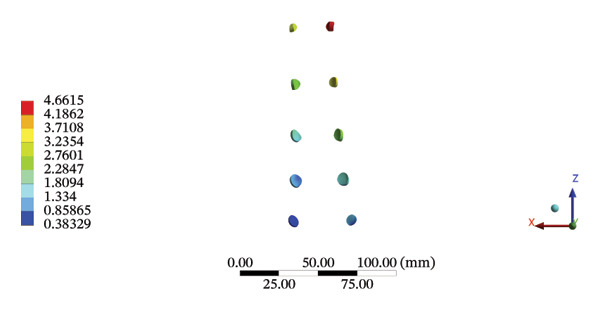
(B)

**Figure:**
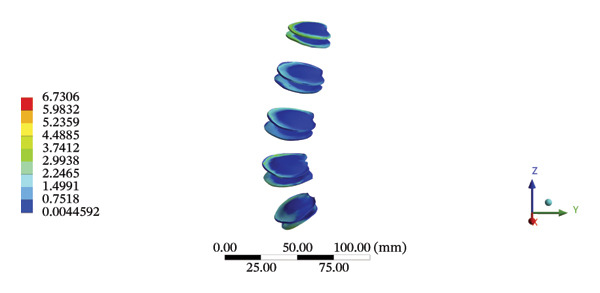
(C)

**Figure:**
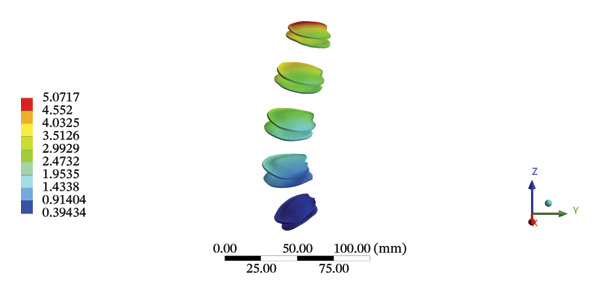
(D)

**Figure:**
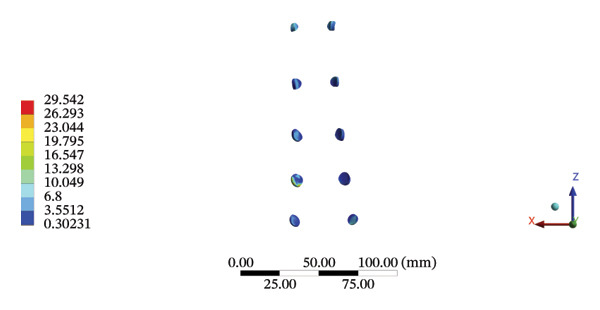
(E)

**Figure:**
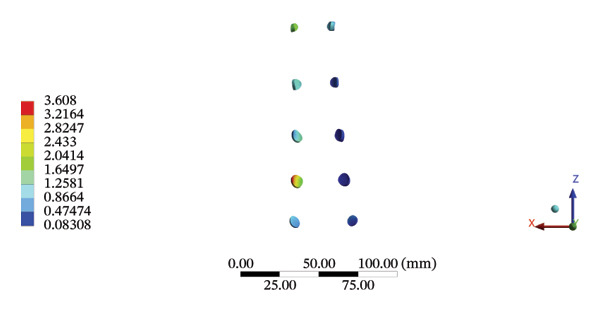
(F)

**Figure:**
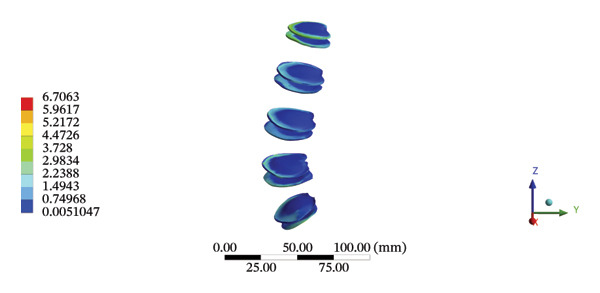
(G)

**Figure:**
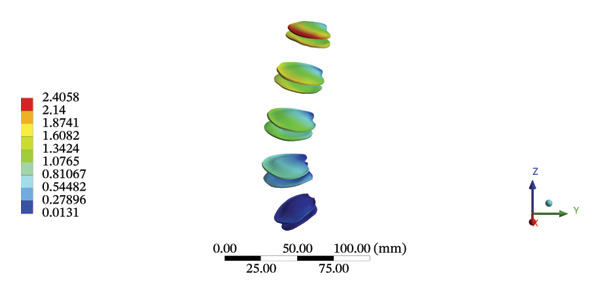
(H)

#### 3.5.2. Pelvic Biomechanics

The maximum stress and displacement at the sacrum were 58.6800 MPa, and 0.3547 mm, respectively, during HSOT. The maximum stress (20.6700 MPa) and maximum displacement (0.1132 mm) were also found at the sacrum during LSOT. The maximum stress and average displacements of other pelvic bone structures during HSOT and LSOT are detailed in Supporting Tables S3 and S4, respectively. Graphical comparisons of the maximum stress and average displacement of the pelvis using HSOT and LSOT are shown in Figure 7.

FIGURE 7Comparisons between the maximum stress and average displacement of the pelvic bone structure using high‐speed oblique Tuina (HSOT) and low‐speed oblique Tuina (LSOT). Notes: (A) shows maximum stress of the pelvic bone structure during HSOT; (B) shows average displacement of the pelvic bone structure during HSOT; (C) shows maximum stress of the pelvic bone structure during LSOT; (D) shows average displacement of the pelvic bone structure during LSOT.

**Figure:**
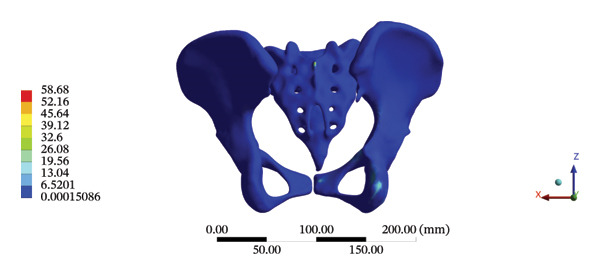
(A)

**Figure:**
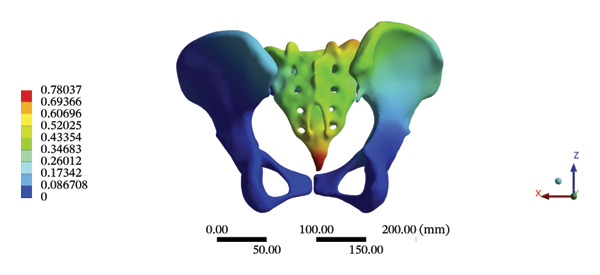
(B)

**Figure:**
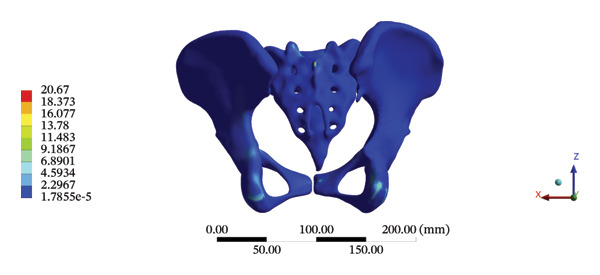
(C)

**Figure:**
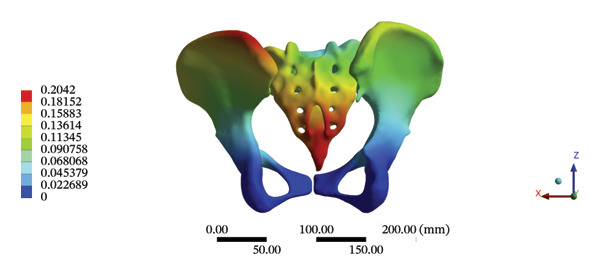
(D)

### 3.6. Lumbar Discs

During HSOT, the maximum stress was found at the left outer layer of the L4‐5 fibrous ring (3.9852 MPa), with an overall average deformation of 1.4324 mm. The maximum stress was also noted at the left L4‐5 nucleus pulposus. The overall average stress reached 0.0244 MPa, while the overall average displacement was 1.3872 mm (Supporting Table S5). During LSOT, the maximum stress was noted at the anterior outer layer of L4‐5 fibrous rings (4.4321 MPa), while the corresponding overall average deformation was 0.4920 mm. Likewise, the maximum stress (0.8753 MPa) was found at the left anterior nucleus pulposus at the L4‐5 level, with an overall average displacement of 0.4552 mm (Supporting Table S6). The graphical comparisons of the maximum stress and average displacement of lumbar discs during HSOT and LSOT are shown in Figure 8.

FIGURE 8Comparison between the maximum stress and average displacement of the lumbar discs structure using high‐speed oblique Tuina (HSOT) and low‐speed oblique Tuina (LSOT). Notes: (A) shows maximum stress of the fibrous ring during HSOT; (B) shows average displacement of the fibrous ring during HSOT; (C) shows maximum stress of the nucleus pulposus during HSOT; (D) shows average displacement of the nucleus pulposus during HSOT; (E) shows maximum stress of the fibrous ring during LSOT; (F) shows average displacement of the fibrous ring during LSOT; (G) shows maximum stress of the nucleus pulposus during LSOT; (H) shows average displacement of the nucleus pulposus during LSOT.

**Figure:**
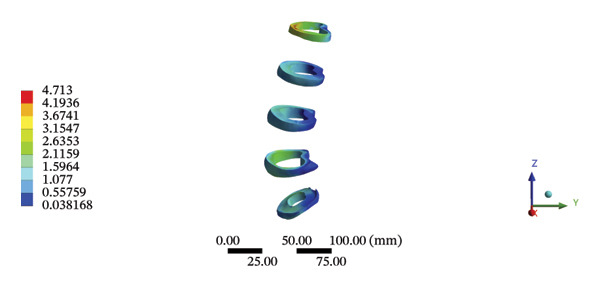
(A)

**Figure:**
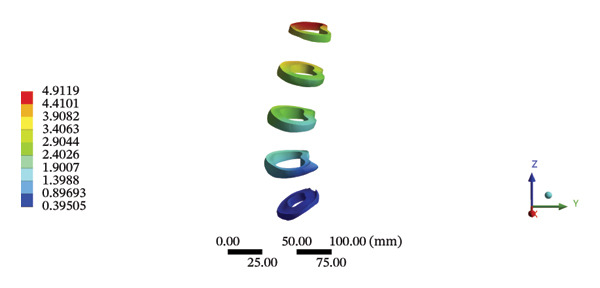
(B)

**Figure:**
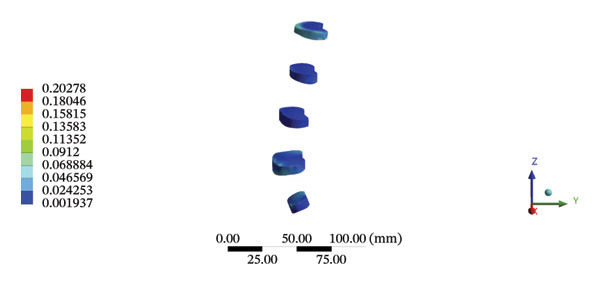
(C)

**Figure:**
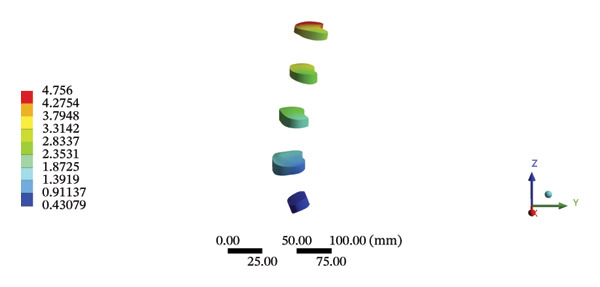
(D)

**Figure:**
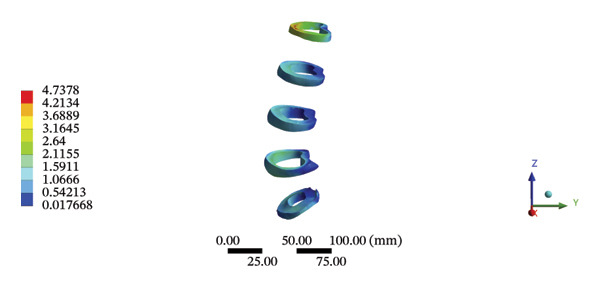
(E)

**Figure:**
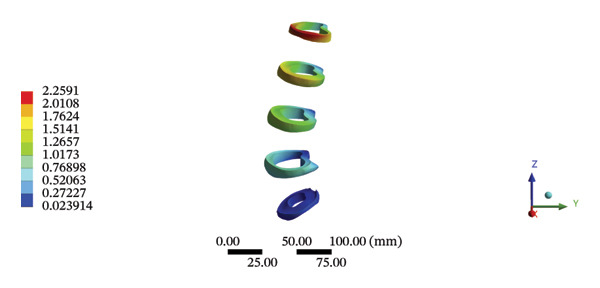
(F)

**Figure:**
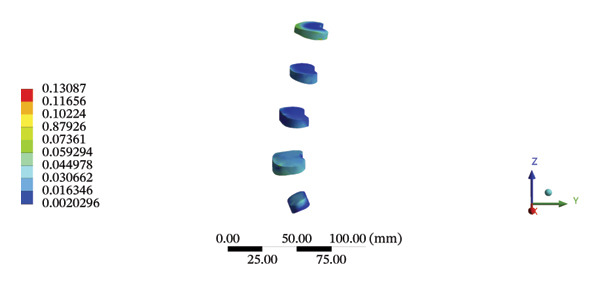
(G)

**Figure:**
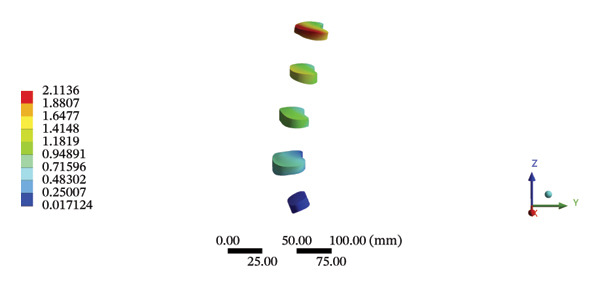
(H)

### 3.7. Lumbar and Pelvic Ligaments

During HSOT, the anterior longitudinal ligament experienced the highest stress (0.0760 MPa), while the maximum displacement was observed at the L4‐5 right intertransverse ligament (2.1747 mm). However, the interspinous ligament underwent the highest stress (0.2780 MPa) during LSOT, whereas the maximal average displacement was found at the L4‐5 left intertransverse ligament (0.9481 mm) (Supporting Tables S7 and S8). HSOT induced the highest stress at the right iliolumbar ligament (0.3551 MPa), and the largest displacement was found at the right iliolumbar ligament (0.9378 mm) in the pelvic region. Conversely, LSOT generated the largest stress on the right sacrotuberous ligament (0.0814 MPa), while the left iliolumbar ligament showed the largest average displacement (0.3971 mm) (Supporting Tables S9 and S10). The graphical comparisons of the maximum stress and average displacement of the lumbar and pelvic ligaments during HSOT and LSOT are shown in Figures 9 and 10.

FIGURE 9Comparisons of the maximum stress and average displacement of the lumbar ligaments using high‐speed oblique Tuina (HSOT) and low‐speed oblique Tuina (LSOT). Notes: (A) shows maximum stress of the lumbar ligaments during HSOT; (B) shows average displacement of the lumbar ligaments during HSOT; (C) shows maximum stress of the lumbar ligaments during LSOT; (D) shows average displacement of the lumbar ligaments during LSOT.

**Figure:**
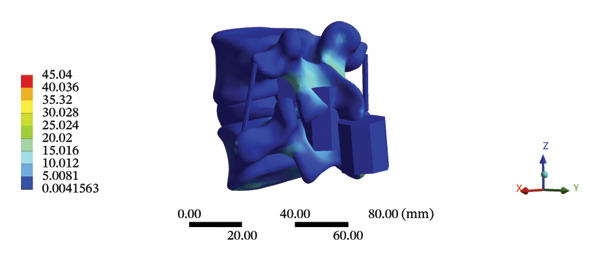
(A)

**Figure:**
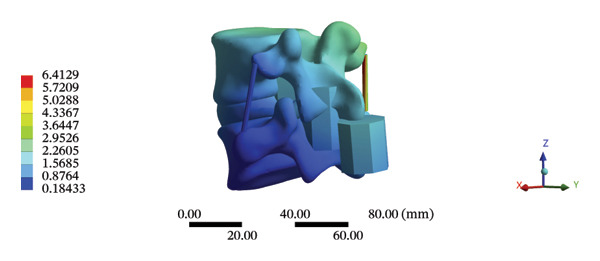
(B)

**Figure:**
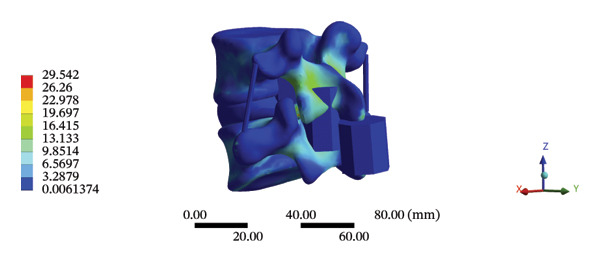
(C)

**Figure:**
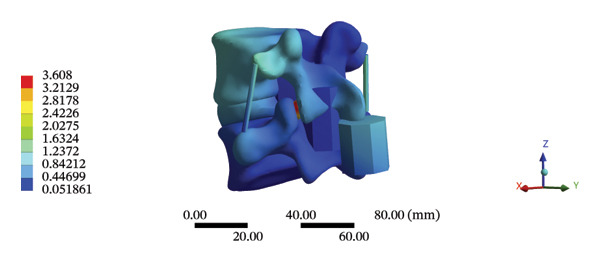
(D)

FIGURE 10Comparison between the maximum stress and average displacement of the pelvic ligaments using high‐speed oblique Tuina (HSOT) and low‐speed oblique Tuina (LSOT). Notes: (A) shows maximum stress of the pelvic ligaments during HSOT; (B) shows average displacement of the pelvic ligaments during HSOT; (C) shows maximum stress of the pelvic ligaments during LSOT; (D) shows average displacement of the pelvic ligaments during LSOT.

**Figure:**
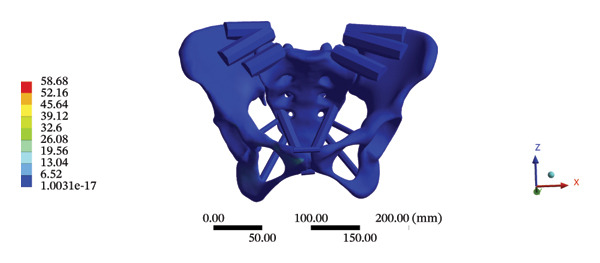
(A)

**Figure:**
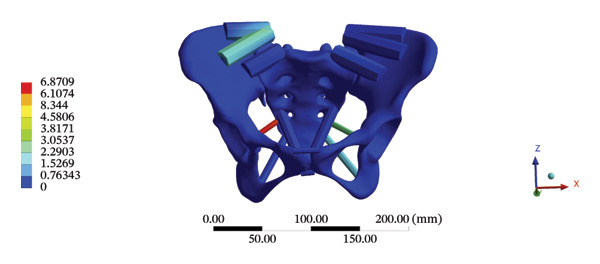
(B)

**Figure:**
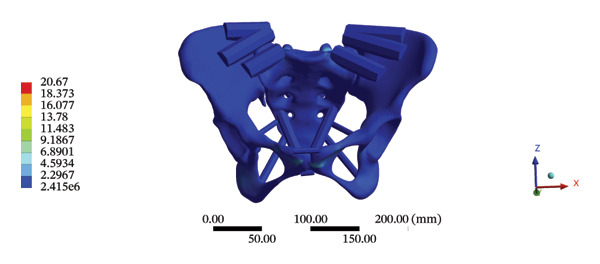
(C)

**Figure:**
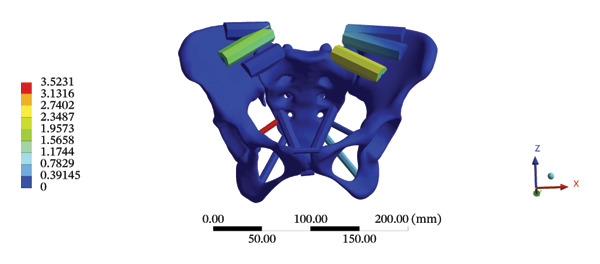
(D)

## 4. Discussion

Lumbar disc herniation is a leading cause of LBP, which ranks first in years lived with disability and sixth in overall disability‐adjusted life years [37, 38]. Tuina may alter the spinal biomechanics (e.g., mobilization of bony structures, alleviating nerve root compression secondary to LDH, reducing paraspinal muscle tone) and promote the absorption of endogenous inflammatory substances [39, 40]. HSOT and LSOT have shown clinical effectiveness in treating individuals with LDH [13]. However, the mechanisms underlying the effects of HSOT and LSOT on the LDH remain unclear. The current study used a 3D FEM to quantitatively analyze the effects of HSOT and LSOT on the stress and displacements of the spinal and pelvic structures, as well as the surrounding ligaments. It quantified the biomechanical effects of HSOT and LSOT on these structures.

Both HSOT and LSOT caused the greatest displacement at the left L4/5 facet joint, although the displacement induced by LSOT doubled that of HSOT. The lumbar disc and bilateral facet joints form the lumbar three‐joint complex connecting adjacent segments [41], and the nerves innervating facet joints originated from the dorsal branch are densely distributed and play a crucial role in pain and proprioception [42, 43]. Research has shown that the degeneration of facet joints is closely related to LBP [44], and the articular cartilage covering the surface of facet joints could reduce friction during joint movement, as well as bear tensile, compressive, and shear loads [45]. Both HSOT and LSOT could modify the stress and strain of facet joints, and fully distract facet joints, which may be one of the biomechanical mechanisms of HSOT and LSOT. However, the greater displacement of the left L4/5 facet joint by LSOT as compared to HSOT might be attributed to the repeated applications of LSOT in clinical practice.

Regarding the mechanical effects of HSOT and LSOT on the pelvis, the maximum stress and displacement of both treatments were found at the sacrum. However, the corresponding values of LSOT were three times smaller than those of HSOT. The sacroiliac joint is an amphiarthrosis, and its front part has synovium that allows slight movement, while the rear part is stabilized by the interosseous ligaments [46]. The usual displacement range of sacrum was less than 1 mm, and its rotation in standing or sitting posture was smaller than 2°. Our displacement data suggested that HSOT (0.06 mm) and LSOT (0.19 mm) did not move the sacroiliac joint beyond the normal range.

Our results revealed that HSOT caused the L4‐5 disc (including annulus fibrosus and nucleus pulposus) to move to the left, anterior, and cephalic directions. On the contrary, LSOT caused L4‐5 lumbar disc to displace in the left, posterior, and cephalic directions. The HSOT generated an impulse to the annulus fibrosus and nucleus pulposus of the L4‐5 disc. The HSOT‐related maximum stress was similar to the findings of a finite element study that investigated the effect of spinal manipulation on a LDH model [26]. However, the displacement of the L4‐5 intervertebral disc in the current study was greater than that (0.45–0.75 mm) induced by an oblique wrench method and the combination of traction, pressing, and oblique pulling on the LDH [31]. It may indicate that HSOT could better induce temporary lumbar disc displacement than other spinal manipulation techniques.

Lumbar ligaments play important roles in maintaining spinal stability. The degeneration of the lumbar structure is associated with the biomechanical changes in lumbar ligaments, especially the reduced stiffness and ultimate strength of the ligamentum flavum, interspinous ligament, supraspinal ligament, and intertransverse ligament [47]. HSOT and LSOT cause considerable displacement of the lumbar ligaments, especially the intertransverse ligaments on both sides. Research has shown that the intertransverse ligament provides minimal stability during physiological movements, but it serves as the main load‐bearing ligament during maximum bending of lumbar lateral flexion, providing support for spinal stability and playing a crucial role in spinal degeneration [48]. Qu et al. conducted anatomical observations and measurements of the intertransverse ligament and found that the average length of the L4‐5 intertransverse ligament was the shortest (1.50 ± 0.20 cm), the average width was the largest (1.04 ± 0.23 cm), and the average thickness was slightly different from other ligaments (0.09 ± 0.04 cm). Therefore, it can be inferred that the L4‐5 intertransverse ligament plays a major role in the stability of spinal lateral flexion. The combination of 1 HSOT and 3 LSOT maneuver can enhance the displacement of lumbar ligaments, particularly the displacement of the left and right intertransverse ligaments.

Prior finite element studies on LDH only used models with two segments without considering any adjacent ligaments or pelvic structures [22, 49]. Given the limitations in the modeling accuracy and scope of the modeling, their conclusions should be interpreted with caution. The current study addressed such gaps by developing a 3D FEM of the lumbar spine and pelvis, containing ligaments, which could better estimate the displacements of relevant bones and ligaments. Moreover, we used CT scan image slide with a 0.5‐mm thickness to construct the FEM, which improved its accuracy. Additionally, we used a research‐grade pressure testing system to collect various mechanical parameters during HSOT and LSOT to inform the FEM, which overcame the shortcomings of some manual therapy‐related FEA studies that entered mechanical information from other biomechanical studies that only collected manual therapy–related mechanical parameters without performing FEA [50, 51]. Thus, we provided a clearer reflection of the real clinical biomechanical mechanisms [32].

This current study has some limitations. First, although muscles and other soft tissues might affect the stability of the lumbopelvic region [52], we did not measure the relevant muscle activity during HSOT or LSOT, which prevented the evaluation of paraspinal muscle responses. Future studies should measure the muscle responses during Tuina so that a better FEM can be constructed. Second, given the difficulty in obtaining cadaveric specimens, the material properties of the spinal structure were estimated based on assumptions. That said, finite element mechanics research is not completely equivalent to human cadaver biomechanics research [53]. It only provided a better understanding of mechanical responses of spinal structures to treatment. Third, the generalizability of our findings was constrained by the narrow pathological spectrum investigated, as this study solely focused on intervertebral discs with Pfirrmann grade‐III degeneration. As such, the mechanical responses of the spine may differ in people with different severities of disc degeneration or LDH [54]. For instance, the size of L4‐5 intervertebral foramen of the L4‐5 disc with severe lumbar degeneration was not significantly changed than those before pushing [30]. Further studies involving individuals with different extents of LDH are warranted in the future. Fourth, due to the relatively simple modeling of spinal soft tissues and the use of linear elastic constitutive relations for all materials included in the FE model, this may not fully reflect the true biomechanical effects of spinal manipulation.

## 5. Conclusions

This study estimated the differential dynamic mechanical response of the lumbar spine during HSOT and LSOT using FEM. Both HSOT and LSOT caused displacements of vertebral bones, intervertebral discs, and lumbar and pelvic ligaments, with the largest displacement of facet joints, intervertebral disc at the L4/5 level. Collectively, HSOT caused larger displacement and stress on most structures compared to LSOT, except that the LSOT‐related displacement of the L4/5 zygapophysial joint doubled that of HSOT. Future studies should determine whether the extent of displacement during the treatment is related to the corresponding clinical improvements.

## Author Contributions

Frank Fan Huang was responsible for the conception of the entire research work, the acquisition and analysis of data, and the drafting of the research content. Ming‐wang Qiu was responsible for the acquisition and analysis of data, as well as the drafting of the research content. Wan‐ming Wu was responsible for drafting the research content and substantially revising it in accordance with relevant requirements. Jia‐jun Liu was responsible for the acquisition and analysis of data. Si‐yi Zhao and Jia‐qi Li were responsible for substantially revising the research content in line with relevant requirements. Man‐qi Lu was responsible for the acquisition and analysis of data. Zhao‐xian Yan was responsible for the acquisition of data. Xian Liu was responsible for the acquisition of data and the quality supervision of the entire research work. Yi‐kai Li was responsible for providing detailed professional revision suggestions and the quality supervision of the entire research work. Arnold Yu Lok Wong was responsible for providing detailed professional revision suggestions and the quality supervision of the entire research work. Zhi‐yong Fan was responsible for providing detailed professional revision suggestions and the quality supervision of the entire research work. All authors agreed to assume personal responsibility for their own contributions. They also ensure that issues related to the accuracy or integrity of any part of the work, even those in which the authors were not personally involved, are appropriately investigated, resolved, and the solutions are documented in the literature.

## Funding

This study was supported by the Lin’s Bone‐Setting Massage School Inheritance (E43611).

## Disclosure

All the listed authors have approved the submitted version (as well as any substantially revised versions concerning the authors’ contributions to the research).

## Ethics Statement

All authors confirm that this experimental protocol was approved by the Institutional Ethics Committee of Guangdong Hospital of Traditional Chinese Medicine (No. YF 2022‐115‐01) and informed consent was obtained from the subject.

## Conflicts of Interest

The authors declare no conflicts of interest.

## Supporting Information

Supporting Tables: Tables S1–S14.

## Supporting information


**Supporting Information** Additional supporting information can be found online in the Supporting Information section.

## Data Availability

The data that support the findings of this study are available from the corresponding author upon reasonable request.
