# Supplementary material for: Biomechanical Comparisons of Two Types of Tuina in Treating Lumbar Disc Herniation: A Finite Element Analysis
Source: Pain Res Manag. 2026 Feb 27;2026:6643204. doi: 10.1155/prm/6643204 (PMC12949095; doi:10.1155/prm/6643204)
Supplement: Supplementary file 1 — Supporting Information Additional supporting information can be found online in the Supporting Information section. [file PRM-2026-6643204-s001.docx]

**Supplemental Tables for “Biomechanical comparisons of two types of Tuina in treating lumbar disc herniation: a finite element analysis”**

Table S1 Material properties of the finite element model

| **Number** | **Materials** | **Elastic modulus（MPa）** | **Poisson ratio** | **Cross-sectional area（mm2）** |
| --- | --- | --- | --- | --- |
| 1 | Cortical bone | 11520 | 0.2 | - |
| 2 | Cancellous bone | 126 | 0.3 | - |
| 3 | Endplate | 32 | 0.25 | - |
| 4 | Anulus fibrosus | 92 | 0.45 | - |
| 5 | Nucleus pulposus | 1 | 0.49 | - |
| 6 | Posterior elements | 3,500 | 0.3 | - |
| 7 | Articular cartilage | 35 | 0.4 | - |
| 8 | Anterior Longitudinal Ligament | 20 | 0.3 | 63.7 |
| 9 | Posterior Longitudinal Ligament | 70 | 0.3 | 314 |
| 10 | Ligamentum flavum | 50 | 0.3 | 314 |
| 11 | Interspinal ligament | 28 | 0.3 | 30 |
| 12 | Supraspinous ligament | 28 | 0.3 | 314 |
| 13 | Intertransverse ligament | 58.7 | 0.3 | 3.6 |
| 14 | Pubic ligament | 10 | 0.3 | 30 |
| 15 | Posterior Sacroiliac Ligament | 13.3 | 0.2 | 100 |
| 16 | Anterior Sacroiliac Ligament | 20.8 | 0.2 | 314 |
| 17 | Lliolumbar ligament | 30 | 0.3 | 314 |
| 18 | Sacrotuberous ligament | 50 | 0.3 | 50 |
| 19 | Sacrospinous ligament | 12.5 | 0.3 | 30 |

Table S2 Mechanical parameters of high-speed oblique Tuina (HSOT) and low-speed oblique Tuina (LSOT)

| **Operation** | **Average impact force(N)** | **Impact time(s)** | **Return wave trough time(s)** |
| --- | --- | --- | --- |
| HSOT 1 | 595.8 | 0.14 | 1.5 |
| HSOT 2 | 575.9 | 0.15 | 1.6 |
| HSOT 3 | 568.3 | 0.16 | 1.4 |
| Average value of HSOT | 580 | 0.15 | 1.5 |
| LSOT 1 | 226.1 | 0.32 | 1.2 |
| LSOT 2 | 219.3 | 0.26 | 1.1 |
| LSOT 3 | 214.6 | 0.28 | 1.3 |
| Average value of LSOT | 220 | 0.30 | 1.2 |

Table S3 Different grid division schemes

| **Grid independence verification** | **Node** | **Grid** | **Overall stress results（MPa）** |
| --- | --- | --- | --- |
| Automatic system division | 130938 | 66791 | 26.256 |
| Mesh size 5.0mm | 154792 | 81435 | 22.188 |
| Mesh size 4.5mm | 178406 | 94423 | 24.264 |
| Mesh size 4.0mm | 211100 | 112545 | 31.34 |
| Mesh size 3.5mm | 260313 | 140099 | 34.12 |
| Mesh size 3.0mm | 332153 | 180262 | 28.603 |
| Mesh size 2.5mm | 450986 | 247121 | 27.024 |
| Mesh size 2.0mm | 675987 | 375880 | 27.534 |

Table S4 Validation of finite element model

| study name | Forward bending and backward extension range of motion | Left and right lateral bending range of motion | Axial rotation activity range |
| --- | --- | --- | --- |
| Pengfei Wen  et al[27] | 7.25° | 7.25° | 3.88° |
| Mingzhi Song  et al[28] | 10.5° | 11° | 4.75° |
| This study | 9.8353° | 9.8445° | 5.1921° |

Table S5 Maximum stress and displacement changes of lumbar spine bone structure using high-speed oblique Tuina

| **Bone structure name** | **Maximum stress (MPa)** | **Average displacement (mm)** |
| --- | --- | --- |
| Right L1/2 articular cartilage | 10.2100 | 4.4531 |
| Right L2/3 articular cartilage | 7.1798 | 3.4215 |
| Right L3/4 articular cartilage | 6.9037 | 2.4792 |
| Right L4/5 articular cartilage | 11.1090 | 1.5667 |
| Right L5/S1 articular cartilage | 5.6587 | 0.4754 |
| Left L1/2 articular cartilage | 11.0820 | 3.2344 |
| Left L2/3 articular cartilage | 7.5547 | 2.4958 |
| Left L3/4 articular cartilage | 6.1232 | 1.7329 |
| Left L4/5 articular cartilage | 10.7720 | 1.1070 |
| Left L5/S1 articular cartilage | 5.6587 | 0.4754 |
| L1 Cortical bone | 33.5150 | 4.2768 |
| L2 Cortical bone | 25.3790 | 3.4423 |
| L3 Cortical bone | 48.1930 | 2.6801 |
| L4 Cortical bone | 20.6320 | 1.8321 |
| L5 Cortical bone | 45.0400 | 1.0169 |
| L1 Cancellous bone | 2.7904 | 4.2375 |
| L2 Cancellous bone | 1.3488 | 3.4984 |
| L3 Cancellous bone | 1.3264 | 2.9874 |
| L4 Cancellous bone | 1.6977 | 1.9146 |
| L5 Cancellous bone | 0.8487 | 1.0048 |
| L1/2 Endplate | 6.7306 | 3.7631 |
| L2/3 Endplate | 3.3520 | 3.0969 |
| L3/4 Endplate | 2.9797 | 2.3772 |
| L4/5 Endplate | 4.2396 | 1.4462 |
| L5/S1 Endplate | 5.9934 | 0.5925 |

Table S6 Maximum stress and displacement changes of lumbar spine bone structure using low-speed oblique Tuina

| **Bone structure name** | **Maximum stress (MPa)** | **Average displacement (mm)** |
| --- | --- | --- |
| Right L1/2 articular cartilage | 10.1600 | 0.8816 |
| Right L2/3 articular cartilage | 7.2681 | 0.4347 |
| Right L3/4 articular cartilage | 5.1251 | 0.2464 |
| Right L4/5 articular cartilage | 9.1214 | 0.1882 |
| Right L5/S1 articular cartilage | 9.5175 | 0.4646 |
| Left L1/2 articular cartilage | 11.0820 | 1.669 |
| Left L2/3 articular cartilage | 7.4508 | 1.0864 |
| Left L3/4 articular cartilage | 7.1353 | 1.0697 |
| Left L4/5articular cartilage | 29.5420 | 2.5362 |
| Left L5/S1 articular cartilage | 7.7330 | 0.7367 |
| L1 Cortical bone | 32.2840 | 1.5565 |
| L2 Cortical bone | 20.8250 | 1.1561 |
| L3 Cortical bone | 12.3510 | 0.8905 |
| L4 Cortical bone | 16.8490 | 0.6135 |
| L5 Cortical bone | 24.5270 | 0.2959 |
| L1 Cancellous bone | 2.3810 | 1.5973 |
| L2 Cancellous bone | 1.0529 | 1.2015 |
| L3 Cancellous bone | 1.2689 | 0.9841 |
| L4 Cancellous bone | 1.5079 | 0.6687 |
| L5 Cancellous bone | 0.6274 | 0.2463 |
| L1/2 Endplate | 6.7063 | 1.5777 |
| L2/3 Endplate | 3.3951 | 1.1520 |
| L3/4 Endplate | 2.6150 | 0.8752 |
| L4/5 Endplate | 3.4362 | 0.4761 |
| L5/S1 Endplate | 3.8015 | 0.1140 |

Table S7 Stress and displacement results of pelvic bone structure using high-speed oblique Tuina

| **Bone structure name** | **Maximum stress (MPa)** | **Average displacement (mm)** |
| --- | --- | --- |
| Right iliac bone | 22.6320 | 0.1921 |
| Sacrum | 58.6800 | 0.3547 |
| Left iliac bone | 4.7088 | 0.0607 |

Table S8 Stress and displacement results of the pelvic structure by low-speed oblique Tuina

| **Bone structure name** | **Maximum stress (MPa)** | **Average displacement (mm)** |
| --- | --- | --- |
| Right iliac bone | 8.5231 | 0.0671 |
| Sacrum | 20.6700 | 0.0113 |
| Left iliac bone | 6.3614 | 0.0892 |

Table S9 Stress and displacement results of the lumbar disc and annulus fibrosus by high-speed oblique Tuina

| **Structure name** | **Maximum stress (MPa)** | **Average displacement (mm)** | **Average displacement of X-axis (mm)** | **Average displacement of Y-axis (mm)** | **Average displacement of Z-axis (mm)** |
| --- | --- | --- | --- | --- | --- |
| L1/2 Annulus fibrosus | 4.7130 | 3.8177 | 1.0190 | -3.6262 | -0.2043 |
| L1/2 Nucleus pulposus | 0.1306 | 3.8703 | 0.9923 | -3.7153 | -0.1774 |
| L2/3 Annulus fibrosus | 2.2855 | 3.0959 | 0.8305 | -2.9338 | -0.2652 |
| L2/3 Nucleus pulposus | 0.0307 | 3.0998 | 0.8521 | -2.9562 | -0.2691 |
| L3/4 Annulus fibrosus | 2.5538 | 2.3890 | 0.7665 | -2.1812 | -0.4344 |
| L3/4 Nucleus pulposus | 0.0530 | 2.3337 | 0.7761 | -2.1416 | -0.4462 |
| L4/5 Annulus fibrosus | 3.9852 | 1.4324 | 0.4081 | -1.2422 | -0.4527 |
| L4/5 Nucleus pulposus | 0.0950 | 1.3872 | 0.4013 | -1.2319 | -0.4278 |
| L5/S1 Annulus fibrosus | 2.9321 | 0.5986 | 0.0631 | -0.5296 | -0.1844 |
| L5/S1 Nucleus pulposus | 0.2027 | 0.5135 | 0.0321 | -0.4798 | -0.4798 |

Table S10 Stress and displacement results of the lumbar disc and annulus fibrosus by low-speed oblique Tuina

| **Structure name** | **Maximum stress (MPa)** | **Average displacement (mm)** | **Average displacement of X-axis (mm)** | **Average displacement of Y-axis (mm)** | **Average displacement of Z-axis (mm)** |
| --- | --- | --- | --- | --- | --- |
| L1/2 Annulus fibrosus | 4.7378 | 1.5307 | 0.9585 | 0.9983 | -0.2567 |
| L1/2 Nucleus pulposus | 0.1308 | 1.4052 | 0.9324 | 0.9102 | 0.9102 |
| L2/3 Annulus fibrosus | 2.2986 | 1.1519 | 0.7587 | 0.7011 | -0.1845 |
| L2/3 Nucleus pulposus | 0.0306 | 1.0934 | 0.7840 | 0.6964 | -0.1845 |
| L3/4 Annulus fibrosus | 1.9786 | 0.8850 | 0.6872 | 0.4088 | -0.1039 |
| L3/4 Nucleus pulposus | 0.0430 | 0.8624 | 0.6946 | 0.4499 | -0.1132 |
| L4/5 Annulus fibrosus | 4.4321 | 0.4920 | 0.36014 | 0.2019 | -0.0526 |
| L4/5 Nucleus pulposus | 0.0875 | 0.4552 | 0.3489 | 0.2435 | -0.0648 |
| L5/S1 Annulus fibrosus | 2.0813 | 0.1170 | 0.0335 | 0.0457 | -0.0344 |
| L5/S1 Nucleus pulposus | 0.1199 | 0.0659 | 0.0060 | 0.0385 | 0.0385 |

Table S11 Stress and displacement results of the lumbar ligaments by high-speed oblique Tuina

| **Ligaments name** | **Maximum stress (MPa)** | **Average displacement (mm)** |
| --- | --- | --- |
| Anterior longitudinal ligament | 0.0760 | 1.5877 |
| Posterior longitudinal ligament | 0.0404 | 1.3737 |
| Ligamentum flavum | 0.0271 | 1.2834 |
| Interspinous ligament | 0.0267 | 1.3260 |
| Supraspinous ligament | 0.0670 | 1.4077 |
| Left L4/5 intertransverse ligament | 0.0041 | 0.7829 |
| Right L4/5 intertransverse ligament | 0.0662 | 2.1747 |

Table S12 Stress and displacement results of the lumbar ligaments by low-speed oblique Tuina

| **Ligaments name** | **Maximum stress (MPa)** | **Average displacement (mm)** |
| --- | --- | --- |
| Anterior longitudinal ligament | 0.0125 | 0.6614 |
| Posterior longitudinal ligament | 0.0668 | 0.2456 |
| Ligamentum flavum | 0.0709 | 0.3202 |
| Interspinous ligament | 0.2780 | 0.5290 |
| Supraspinous ligament | 0.0985 | 0.6414 |
| Left L4/5 intertransverse ligament | 0.0534 | 0.9481 |
| Right L4/5 intertransverse ligament | 0.0539 | 0.4433 |

Table S13 Stress and displacement results of ligaments of the pelvis by high-speed oblique Tuina

| **Ligaments name** | **Maximum stress(MPa)** | **Average displacement(mm)** |
| --- | --- | --- |
| Pubic ligament | 0.0034 | 0.0085 |
| Right sacrospinous ligament | 0.0836 | 0.2248 |
| Right sacrotuberous ligament | 0.0397 | 0.2525 |
| Right posterior sacroiliac ligament | 0.3551 | 0.4152 |
| Right anterior sacroiliac ligament | 0.2839 | 0.3978 |
| Right liolumbar ligament | 0.3367 | 0.9378 |
| Left sacrospinous ligament | 0.0162 | 0.2141 |
| Left sacrotuberous ligament | 0.0032 | 0.2587 |
| Left posterior sacroiliac ligament | 0.1877 | 0.2857 |
| Left anterior sacroiliac ligament | 0.0704 | 0.2282 |
| Left liolumbar ligament | 0.0854 | 0.2804 |

Table S14 Stress and displacement results of ligaments of the pelvis by low-speed oblique Tuina

| **Ligaments name** | **Maximum stress (MPa)** | **Average displacement (mm)** |
| --- | --- | --- |
| Pubic ligament | 0.0224 | 0.0293 |
| Right sacrospinous ligament | 0.0295 | 0.1047 |
| Right sacrotuberous ligament | 0.0814 | 0.0927 |
| Right posterior sacroiliac ligament | 0.0032 | 0.1218 |
| Right anterior sacroiliac ligament | 0.0153 | 0.0677 |
| Right liolumbar ligament | 0.0621 | 0.2425 |
| Left sacrospinous ligament | 0.0111 | 0.0999 |
| Left sacrotuberous ligament | 0.0297 | 0.0897 |
| Left posterior sacroiliac ligament | 0.0247 | 0.1444 |
| Left anterior sacroiliac ligament | 0.0177 | 0.1106 |
| Left liolumbar ligament | 0.0707 | 0.3971 |
